# BOLD response delays represent local cortical processing

**DOI:** 10.1093/cercor/bhag040

**Published:** 2026-04-22

**Authors:** Sébastien Proulx, Reza Farivar

**Affiliations:** Department of Ophthalmology & Visual Sciences, McGill University, Montreal General Hospital, Room L7-213, 1650 Cedar Avenue, Montréal, Québec, H3G 1A4, Canada; Research Institute of McGill University Health Centre, Montreal General Hospital, Room L7-213, 1650 Cedar Avenue, Montréal, Québec, H3G 1A4, Canada; Department of Ophthalmology & Visual Sciences, McGill University, Montreal General Hospital, Room L7-213, 1650 Cedar Avenue, Montréal, Québec, H3G 1A4, Canada; Research Institute of McGill University Health Centre, Montreal General Hospital, Room L7-213, 1650 Cedar Avenue, Montréal, Québec, H3G 1A4, Canada

**Keywords:** BOLD, cross-orientation suppression, fMRI, hemodynamic response function, V1

## Abstract

A number of studies showed that stimulus or task conditions can alter the shape of the hemodynamic response (HR). Contrary to variations across brains and brain regions, vascular factors alone cannot account for within-voxel HR waveform variations. Instead, different neuron types may contribute differently to shaping the HR, suggesting that beyond detecting neural activations, measurements of stimulus- or task-specific HRs could inform on the nature of underlying neural processes. To assess this hypothesis, we measured HR apparent delays to oriented visual stimuli with 1 mm and 1-s resolution Blood Oxygenation Level Dependent (BOLD) functional MRI (fMRI) in healthy humans. As expected, decoding V1 patterns of HR amplitudes allowed robust cross-validated predictions of stimulus conditions, ie two orthogonal gratings and an overlay of the two. More interestingly, this was also true using patterns of HR delays alone, and predictions using both delay and amplitude information outperformed those using amplitude alone. Finally, while all stimuli evoked similar V1-averaged HR amplitudes, the overlay stimulus’ HR waveform lagged ~180 ms behind that of grating stimuli. We interpret this increased HR delay as reflecting different neural computations, here more cross-orientation suppression with overlay stimuli, and conclude that neurally relevant information can be obtained from the HR waveform in addition to its commonly used amplitude.

## Introduction

The hemodynamic response (HR) amplitude in functional MRI (fMRI) is the most relied upon noninvasive proxy for increases in local neuroelectric and neurometabolic activity (Logothetis 2002; Yacoub et al. 2008; Kim and Ogawa 2012), but it is now well appreciated that relating HR amplitude to neural excitation is an oversimplification—HRs can be triggered by optogenetic activation of either excitatory pyramidal neurons *or* inhibitory interneurons in animal models (Anenberg et al. 2015; Uhlirova et al. 2016b). While the HR shape as a whole reflects complex blood flow, volume, and oxygenation dynamics (Buxton et al. 1998; Kim and Ogawa 2012), components of the HR shape are related to engaged neural processes (Bartolo et al. 2011; Farivar et al. 2011; Uhlirova et al. 2016a; Uhlirova et al. 2016b; Havlicek et al. 2017). Because the vast majority of fMRI studies are focused on relating amplitude changes to specific conditions under manipulation, variations in the HR shape are usually either ignored or modeled out as a nuisance variable (Boynton et al. 1996), despite some evidence that HR dynamics are potentially related to neural processes (Shmuel et al. 2002) and more recently, that there may be cortical depth-related (Siero et al. 2011) and stimulus × depth interaction effects as well (Kashyap et al. 2018). The underlying assumption served by this exclusionary approach is that HR shape features, such as delay, are uninformative—that they have no information to contribute regarding either neural representations or processing of information related to the conditions under investigation.

But features such as delay may contain additional/complementary information. HRs triggered by visual stimuli of varying contrast (Thompson et al. 2014; Chen et al. 2021) or presented alone vs spatially overlaid (Bartolo et al. 2011; Farivar et al. 2011) exhibit varying delays despite being measured within the same patch of brain tissue where the possible effect of purely vascular dynamics is removed (but neural suppression is modulated). Upon visual presentation of a plaid overlay of orthogonal gratings—inducing cross-orientation suppression (Sengpiel and Vorobyov 2005)—compared to gratings presented alone, Bartolo et al. (2011) observed larger HR delays accompanied by reduced γ-band local field potential (γ-LFP) and increased spiking activity in nonhuman primates. Farivar et al. (2011) showed in amblyopic patients—a population that typically experiences interocular suppression—that HRs to visual stimulation of the pathologically suppressed eye exhibit an unusually delayed HR compared to stimulation of the normal eye, a delay that was further deepened by the use of dichoptic stimuli designed to maximize interocular suppression. These reports jointly suggest that HR dynamics—particularly delay—may reflect specific processes such as cortical suppression.

An important approach to understanding the relationship between suppressive neural activity and HR shape has been to directly stimulate populations of neurons with optogenetics, but relating activity of inhibitory (eg parvalbumin-positive) interneurons to HR dynamics such as delay or polarity has yielded inconsistent results (Dahlqvist et al. 2020; Lee et al. 2021; Vo et al. 2023) and as summarized by Vo et al. (2023)—slow dilation under α-chloralose anesthesia (Dahlqvist et al. 2020) and isoflurane (Lee et al. 2021) and constriction under awake, ketamine/xylazine, and isoflurane conditions (Lee et al. 2021), with a recent report showing important differences between anesthetized and awake states (Vo et al. 2023). Taken together, while animal studies have the potential for elucidating the precise contribution of different cell types to HR dynamics, their translation to the more typical human fMRI study with natural sensory stimulation is currently still limited.

If HR delay reflects an aspect of neural suppression, as suggested by the works discussed above, then HR delay estimates ought to contain process-dependent information. When this suppression is ubiquitous for a specific sensory representation, such as the case of lateral interactions among orientation columns in V1, then spatial patterns of HR delay ought to contribute additional information regarding the signal (stimulus orientation) being represented.

In this study, we tested this hypothesis using high-resolution (1 mm, 1-s) noninvasive BOLD fMRI measures at 3T in the human early visual cortex, which we stimulated with oriented gratings and cross-oriented plaid overlay stimuli to evoke different levels of inhibitory interactions between cortical orientation columns/channels (Hubel and Wiesel 1974; Morrone et al. 1987; Deangelis et al. 1992; Sengpiel and Vorobyov 2005; Angelucci and Bressloff 2006; Meese and Baker 2013). The high resolution was sought to minimize partial volume effects, and the short acquisition was sought to increase our temporal resolution for estimating hemodynamic delays.

We used a periodic stimulus presentation to reduce the complex temporal waveform of the HR to a simple sinusoid and used the delay of this sinusoidal HR as an unspecific but sensitive proxy for HR waveform changes. We adopted a cross-validated multivariate approach to decode cortical patterns of apparent HR delays (Haynes and Rees 2005; Kamitani and Tong 2005), pooling relevant signals across voxels and maximizing sensitivity. Results show that apparent HR delays are sufficient—independently of HR amplitudes—to discriminate BOLD responses evoked by the plaid overlay stimuli from those evoked by single gratings. Moreover, different stimulus orientations produced different cortical patterns of response amplitude, whereas inducing cross-orientation suppression with overlaid stimuli (Angelucci and Bressloff 2006) lengthened the apparent delay of the V1 response but left amplitude unaffected. We conclude that different neural processes in brain tissue showing similar activity levels can affect the shape of the BOLD HR. This opens a novel way to investigate the computational function of active brain tissues, noninvasively with widely available MRI scanners.

## Materials and methods

### Participants

Six (including author S.P.) healthy human adults (1F, 5 M; mean age: 30.8 yrs; age range: 19 to 47) with normal or corrected-to-normal vision participated in this study. Informed consent was obtained from all participants, and the protocol was approved by the Research Ethics Board of the Montreal Neurological Institute (NEU-13-043).

### Stimuli

**Figure 1:**
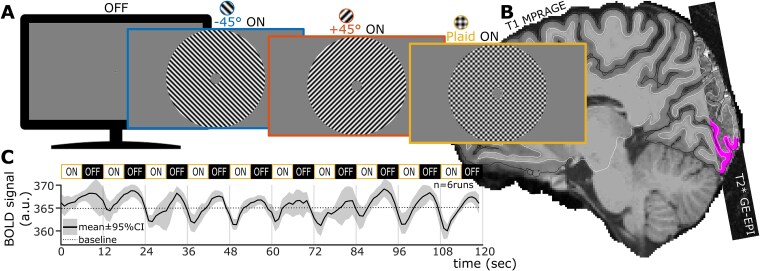
Experiment overview. A) Stimuli presented during OFF (fixation only) and ON (fixation + contrast-reversing grating or plaid overlay) periods. B) Example BOLD functional (T2*-weighted) and anatomical (T1-weighted) images, coregistered using boundary-based registration. Gray matter inner and outer boundaries outlined in dark and white, respectively. Purple overlay shows the V1 gray matter ROI. C) Example BOLD timeseries averaged across the ROI (no spatial feature selection applied) and across runs of the plaid stimulus condition. Data from participant 03sk. Confidence intervals derived from 8,192 bootstrap resamples with replacement.

The stimuli used are shown in Fig. 1A. Sinusoidal grating and plaid stimuli were displayed to participants against a mean luminance gray background through a coil-mounted mirror using a gamma-calibrated MRI-compatible LCD monitor (3-D BOLD Screen, Cambridge Research Systems Ltd) positioned at the back of the MRI bore.

Stimuli in the ON periods of the fMRI stimulus design consisted of stationary sinusoidal gratings or a plaid overlay (2-cpd spatial frequency) with contrast reversal following a square-wave function (8 Hz temporal frequency). Participants viewed the stimuli over a mean luminance background, through an annular aperture centered on fixation (0.7dva-diameter concentric pattern) and spanning from 0.75 to 7dva eccentricity. Grating stimuli were full contrast and orthogonally oriented at ±45° relative to vertical. The plaid stimulus was composed of an overlay of the half-contrast orthogonal gratings, matching root mean squared (RMS) contrast between grating and plaid stimuli. During the OFF periods of the fMRI stimulus design, only fixation over the mean luminance gray background was presented. Between MRI runs, the monitor showed only the mean luminance gray background to minimize adaptation effects.

### MR imaging

We sought to acquire very-high-resolution functional images (ie 1 mm isotropic voxels) to minimize partial volumes—unresolved veins and tissue boundaries decrease the neural specificity of a voxel’s signal. Increased resolution comes at a cost to signal-to-noise ratio (SNR), increased transient (TR) acquisition time and increased echo-planar imaging (EPI) distortions in the phase-encode direction. We mitigated the SNR loss by using a custom 32-channel posterior-only radio-frequency (RF) coil that doubles the SNR in the occipital region (Farivar et al. 2016). We maintained acquisition time within our target TR = 1 s by focusing on a small stack of imaging slices over the occipital pole (Fig. 1B). We acquired extra EPI images with the phase-encoding direction reversed to allow for off-line correction of EPI distortions using the up-down method (Andersson et al. 2003; Holland et al. 2010).

Each MR session lasted ~ 1 h in a 3T Siemens Tim Trio scanner using the body coil for RF transmission. RF signal reception used the Siemens 32-channel full-head coil for whole-head T1-weighted anatomical scans (motion-corrected MEMPRAGE; TR = 2.3 s; TE = 1.74, 3.60, 5.46, and 7.32 ms; TI = 1,260 ms; FA = 7°; GRAPPA 2, 32 ref. line; matrix size = 256 × 256; voxel size = 1 mm × 1 mm; 176 sagittal 1 mm slices; BW = 651 Hz/Px; RMS across echoes) and a custom-built, occipital-cortex dedicated, 32-channel coil array (Farivar et al. 2016) for all other acquisitions, including TRUFISP localizer scans (31 sagittal slices; 1.3 × 1.0 mm in-plane resolution; FOV 250 mm; 4 mm slice thickness with 20% gap; TR 4.6 ms; TE 2.3 ms; FA 37°). Functional acquisitions consisted of 15 to 21 runs of 120 BOLD fMRI high-resolution volumes (1 × 1 × 1 mm^3^ GE-EPI; 13 oblique coronal slices; 10% gap; 128 mm FOV; TR 1,000 ms; TE 33 ms; Echo spacing 1.05 ms; Left–Right phase-encoding direction; GRAPPA = 3 with 126 reference lines; FA = 33°; 4 dummy volumes acquired prior to scanner trigger of the stimulus sequence then discarded). A low flip angle was used to minimize physiological noise (Gonzalez-Castillo et al. 2011). Additional shorter 10-volume functional runs were acquired in the reversed phase-encoding direction at the beginning and end of each session for correction of EPI spatial distortions. To minimize head motion, a bite bar was used for three participants and foam padding for the other three.

Each subject underwent two MRI sessions separated by a ~1-h break. In the first session, functional runs were manually prescribed to cover the tip of and ~0.5 mm beyond the occipital pole with slices roughly perpendicular to the calcarine sulcus (see Fig. 1B), based on the anatomical localizers. In the second session, functional runs’ prescription was matched to that of the first session using Siemens’ auto-align routine with visual confirmation.

### Functional MRI design

During each 120-s functional run, stimuli were presented in 10 consecutive 6 s-ON–6 s-OFF cycles (Fig. 1C). Only one of the three stimulus conditions, namely, −45° grating, +45° grating, or plaid, was presented per run, in an order randomized within three consecutive runs. This three-run sequence was repeated 5 to 7 times per session per participant, with ~10- to 30-s rests between each run.

To maintain alertness, participants performed a simple attentional fixation task throughout each run, producing button-press reports of contrast reversals of the concentric fixation pattern (Fig. 1A; random reversal delays drawn from a flat distribution between 1 and 9 s, on average 26.67 reversals per 120-s run). Performance was not used for analysis.

### MRI preprocessing

#### Functional volume preprocessing

Functional volumes preprocessing used AFNI tools (https://afni.nimh.nih.gov/), starting with slice timing correction and followed by a series of spatial transformations that were combined and applied in one interpolation step to minimize spurious spatial smoothing. These spatial transformations included, chronologically, (i) within-session motion correction, (ii) EPI distortion correction, and (iii) between-session registration.

Within- and between-session registration used AFNI’s 3dvolreg function. EPI spatial distortion correction used the plus–minus nonlinear registration method (AFNI’s 3dQWarp function; Andersson et al. 2003, Holland et al. 2010), aligning EPI images and reversed phase-encoding EPI images only along the phase-encoding axis. Distortions were estimated as such for each separate fMRI session after manually masking out nonbrain voxels. Finally, we used the distortion-corrected images, again masking out nonbrain voxels, to estimate the between-session registration. All spatial correction estimates (ie motion and distortion corrections and between-session registration) were combined and applied to the slice timing–corrected images in one interpolation step using 3dNwarpApply. No spatial smoothing was applied.

Each run timeseries was expressed as %BOLD relative to the run mean, and all further analyses were performed on %BOLD.

#### Retinotopic atlas registration and V1 ROI

We obtained estimates of retinotopy for individual subjects through the registration of a probabilistic retinotopic atlas (Benson et al. 2012) to each participant’s own functional volume space. Brain surface reconstruction from T1-weighted anatomical scans used Freesurfer’s analysis pipeline (http://surfer.nmr.mgh.harvard.edu/). The atlas’ brain surface was registered to each participant’s brain surface using Freesurfer’s tools for nonlinear surface–based registration, following Benson et al. (2012), https://cfn.upenn.edu/aguirre/wiki/public,retinotopy_template).

We then estimated each participant’s functional-to-anatomical registration using boundary-based volume registration (Greve and Fischl 2009). Using the reverse of the estimated functional-to-anatomical registration, we projected the atlas-based retinotopy from each participant’s own surface-based anatomy to their functional volume space.

This allowed the definition, in the native acquisition space of the functional images, of a V1 ROI spawning the full thickness of the cortical gray matter—any voxel intersecting the volume between the white matter and pial surfaces was included, and layer location was not analyzed. This ROI excluded voxels from the first and last slice to avoid partial volumes, after motion correction, with regions out of the imaging field of view. The atlas-based retinotopy also served as priors for the empirical estimation of the cortical representation of the visual stimuli (see section Cortical Representation of the Visual Stimuli).

### Analysis

#### Sinusoidal response vector estimation

**Figure 2:**
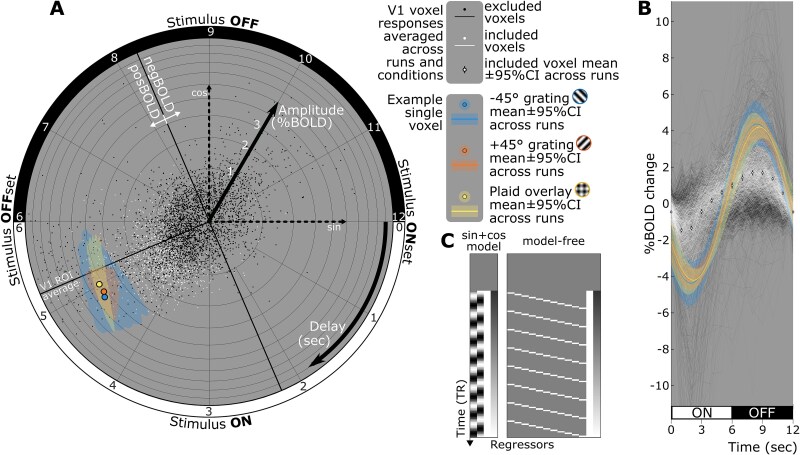
Example single-subject and single-voxel responses. A) Two-dimensional representation of sinusoidal response vectors (sin + cos model). Each white or black dot represents the tip of a single-voxel response vector, with an origin at coordinate [0, 0] of the [sin, cos] Cartesian plane (dashed arrows). Vector length represents half the sinusoidal BOLD response peak-to-peak amplitude, and vector angle represents its phase (positive peak time minus 3 s) relative to stimulus onset. Included voxels (see section spatial feature selection) are shown in white, excluded V1 voxels in black. Also shown is the phase of the V1-averaged response (thin radial dark line) and, orthogonal to that delay, the limit between positive (posBOLD) and negative BOLD (negBOLD) responses. Colored circles and shadings represent an example single-voxel showing stimulus-condition-specific responses concordant with the group-level effect. B) Same data as in A), now expressed as signal change across time through the stimulus cycle, ie the hemodynamic response (HR). Model-free HRs are shown for single voxels (thin white and dark traces) and averaged across included voxels (solid white circles). Stimulus-condition-specific sinusoidal fit to the example single-voxel is shown in colored traces and shadings. C) Design matrices used for fitting BOLD timeseries. For the sin + cos model, the first pair of regressors are the sine and cosine regressors of interest and the second pair is the baseline and signal drift nuisance regressors. For the model-free matrix, the regressors of interest are the first 11. The gray zone corresponds to the censored time points from the first two stimulus cycles. Data from participant 03sk. Confidence intervals derived from 8,192 bootstrap resamples with replacement.

The ON–OFF cyclical pattern of visual stimulation was designed to reduce the BOLD response to a simple sinusoidal shape that could be described with only two parameters: response amplitude and delay. Those parameters are conveniently estimated through the linear fit of a pair of sine and cosine regressor functions (Fig. 2C) matching the 12-s stimulus cycle length. The fit coefficients form a response vector best represented in a 2D complex plane (Fig. 2A), where the real (*x*-axis) and imaginary (*y*-axis) coordinates correspond respectively to the sine and cosine fits. The length and angle of vectors (single dots and circles in Fig. 2A) correspond, respectively, to half the peak-to-peak amplitude and to the phase delay of the stimulus-driven responses.

In a linear model of a voxel’s single-run BOLD timeseries (Fig. 2C), we included sine and cosine regressors of interest along with constant baseline and linear drift nuisance regressors. Fitting used ordinary least-squares regression (OLS), censoring the first 24 timepoints corresponding to the first two stimulus cycles (top gray section in Fig. 2C).

#### Model-free hemodynamic response estimation

For visualization purposes only, the actual shape of the BOLD response to the ON–OFF stimulus cycle was also estimated (Fig. 2B). The sine and cosine regressors in the linear model described above were replaced with 11 delta function regressors modeling signal amplitude from the 2nd (*t*_0 + 1_) to the 12th (*t*_0 + 11_) functional volume into the 12-s stimulus cycle (Fig. 2C). Constant baseline and linear drift regressors were left unchanged. The signal at the first (*t*_0_) volume was implicitly modeled by the baseline. We reconstructed the HRs (Fig. 2B) from *t*_0_ to *t*_0 + 11_ into the stimulus cycle using the fit coefficients (Fig. 2C).

For display purposes only, the interindividual variability in HR amplitude and delay was removed, thereby highlighting stimulus condition effects (Fig. 5B) and interindividual variation in waveform (Supplementary Fig. 4). Dividing the zero-centered HRs of each participant by the length of their respective condition-averaged sinusoidal response vector and multiplying by the length of the group-average response vector effectively removed HR amplitude variations. Delay variations were removed by cubic interpolation of each participant’s HRs on time axes shifted according to the angle difference of the participant’s response vector relative to the group.

#### Spatial feature selection

##### A. Cortical representation of the visual stimuli

**Figure 3:**
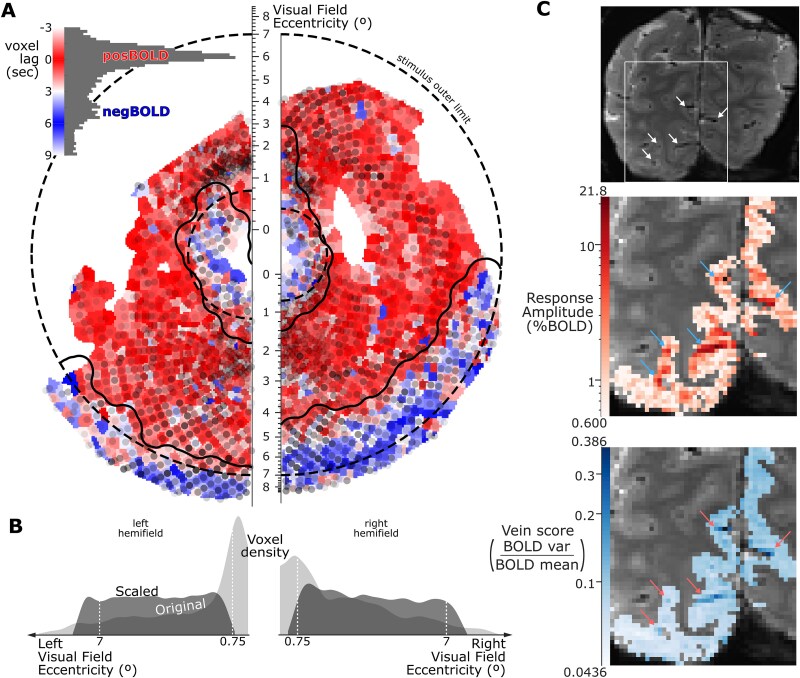
Overview of spatial feature selection. A) Voxel response polarity histogram (inset) and map in a warped visual-field space. Inner and outer dashed lines show the inner and outer limits of the stimulus field of view. Solid lines show the limits of the cortical representation of the stimulus field of view as conservatively estimated from the positive to negative BOLD response transition zones. The transparent dark overlay shows voxel density in warped visual-field space. B) Voxel density (voxels per unit visual-field area) as a function of eccentricity in the original (darker overlay) and warped (lighter overlay) visual-field space. C) BOLD signal characteristics of large veins. White arrows in the top panel show large veins resolved as low signal points or streaks in our 1 × 1 × 1 mm^2^-resolution functional images. The same veins are shown magnified with blue arrows in the middle panel as regions of large BOLD responses, and with red arrows in the lower panel as regions of large signal variability. Data from participant 03sk.

To select voxels within the V1 cortical representation of the stimulus field of view, we relied on the probabilistic retinotopic atlas registered to each participants cortical folding (see section Retinotopic Atlas  Registration and V1 ROI), further refined functionally using the boundaries between posBOLD and negBOLD foci expected, respectively, within and surrounding the stimulus representation (Fig. 3A and B; Smith et al. 2004; Shmuel et al. 2006; Wade and Rowland 2010). Specifically, the polarity of a voxel’s sinusoidal (12 s-period) response (Fig. 3A, inset) was considered positive (posBOLD) when its phase lagged within −π/2 to π/2 (−3 to 3 s) of the ROI-averaged response (Fig. 2A), and negative (negBOLD) otherwise (6 to 9 s). This was visualized in a visual-field space for each participant’s hemifield (Fig. 3A), where the eccentricity axis was warped to uniformize voxel density (Fig. 3B), account for cortical magnification and better visualize boundaries (see Supplementary Fig. 1 for details). Finally, the stimulus’ retinotopic boundaries were refined through a heuristic involving iterative smoothing of the response polarity map and contour extraction, inflation, merging, and selection (see Supplementary Fig. 1 for details), yielding a conservative definition of the stimulus’ cortical representation (Fig. 3A).

##### B. Stimulus-driven voxels

We identified stimulus-driven voxels as those showing non-random response vectors across runs. The 2D coordinates of response vectors were entered as two dependent variables in a multivariate ANOVA for repeated measures implemented in an adaptation of the manova.m function from MATLAB’s Statistical Toolbox. The statistical model included the repeated-measure factor of stimulus condition and the intercept, the latter effectively testing whether the mean response vector differed from the 0-length null vector. Voxels with a significant main effect of the intercept (α = 0.05) were selected without correction for multiple comparisons.

##### C. Nonvein voxels

Large vein voxels are known to show low mean signals (Fig. 3C, white arrows in the top panel), large stimulus-driven BOLD responses (Fig. 3C, blue arrows in the middle panel), and high signal variability (Olman et al. 2007; Kay et al. 2019). We therefore computed the ratio of the standard deviation of a voxel’s detrended absolute BOLD (not %BOLD) timeseries over its baseline (Fig. 3C, bottom panel) as a commonly used metric of the likeliness of a voxel containing a large vein. A threshold was defined as the vein likeliness metric at the 80th percentile of voxels having passed feature selection steps A and B. Voxels below this vein likeliness threshold were selected.

##### D. Most discriminant voxels

The sensitivity of a voxel to stimulus conditions was evaluated as the Hotelling’s *T*^2^ statistics of the main effect of stimulus condition in the multivariate ANOVA described in feature selection step B. A threshold was defined as the *T*^2^ at the 20th percentile of voxels having passed feature selection steps A through C. Voxels above this *T*^2^ threshold were selected.

#### Support vector machine training

We used the spatial patterns of BOLD responses’ delay, amplitude, or both to train a Support Vector Machine (SVM) algorithm for the pairwise classification of stimulus conditions, independently for each participant, session, and stimulus condition pair. The training data consisted of a *n* × *p* complex-valued matrix containing the sinusoidal response vectors, with *n* runs as samples and *p* voxels as features. Mainly to minimize phase wrap, the matrix was first normalized by rotating and scaling response vectors voxel-by-voxel to a mean of 0-angle and length 1.

For classification based on response delay only, we replaced response vectors in the data matrix by their angle. For classification based on response amplitude only, we replaced the response vectors by their length. For classification based on both response delay and amplitude, we concatenated the real and imaginary parts of the complex-valued *n* × *p* matrix of response vectors into a real-valued *n* × 2*p* matrix. This latter approach was inspired by Bouboulis and Theodoridis (2011) and Bouboulis et al. (2015) to allow the training of standard algorithms with complex-valued data.

After further *z*-scoring voxel by voxel, all model training used the linear C-SVM classifier algorithm from the LiBSVM-3.24 library with default parameters (Chang and Lin 2011).

#### Cross-validation between sessions

We leveraged our two-repeated-session design to avoid circular inference. Only anatomical information bridged the two sessions: through registration of the functional spaces and of the probabilistic retinotopic atlas. Response vector estimation, spatial feature selection, and SVM training in one session were strictly independent of the other sessions. Only the trained models crossed from the “train” session to the other “test” session for computing the classification performance metric that was then averaged across sessions for group-level inference statistics. Similarly, when averaging response vectors and time courses across voxels, each session used the voxel selection derived from the other session.

We used the Area Under the receiver operating characteristic (ROC) Curve (AUC) to assess classification performance. It is interpreted in the same way as a percent correct accuracy estimate, ie ranging between 0 and 1 with a chance level at 0.5, but has the advantage of being threshold insensitive.

### Statistics

Significance of AUC was determined against a null distribution empirically derived from 8,192 permutations of condition labels within each 3-run repetition. We applied this permutation of labels at an early stage, before response vector estimation. An actual AUC larger than 95% of its corresponding permuted AUCs was deemed significantly (uncorrected one-sided *P* < 0.05) above the 0.5 chance level. Repeated-measures ANOVAs, Student *t*, and signed-rank tests, and Pearson’s and Spearman’s correlations all used α = 0.05.

We derived all confidence intervals from 8,192 bootstrap resamples with replacement. For single-participant statistics, each resample contained the same number of runs as the original sample. For group statistics, each resample contained *n* = 6 participants. Bivariate confidence intervals used the probability density map—based on a normal kernel function—of resampled means, where we lowered a probability threshold to define a contour that grows around the true mean until encompassing 95% of the resamples. This contour was taken as the credible interval, as termed in Bayesian statistics, but here referred to as the confidence interval for simplicity.

## Results

### Data overview and voxel selection

Figures 1–3 summarize data from one representative participant, sk03. The HRs (Fig. 2B) extracted from voxels within the V1 gray matter ROI (Fig. 1B) did follow the expected sinusoidal shape, albeit with a positive lobe appearing wider than the negative one (Supplementary Fig. 4). The compact representation of the participant’s HRs as sinusoidal response vectors on a polar plot (Fig. 2A) showed an ROI-averaged apparent hemodynamic delay of 5.2 s (Fig. 2A, dark line in the lower-left quadrant; phase delay between the sinusoidal function fit and the stimulus presentation’s square-wave function). This delay ranged from 3.6 to 5.6 s across sessions and participants (mean 4.9 s), consistent with an expected long and variable hemodynamic delay of vascular origin (Handwerker et al. 2004; Proulx et al. 2014).

Voxel responses showed a bimodal distribution of phases (inset in Fig. 3A and Supplementary Fig. 2), where several voxels have a phase opposite to the main hemodynamic delay (responses above the solid dark diagonal in Fig. 2A). In our example participant, 33% of ROI voxels showed this characteristic consistent with negative BOLD responses (35% on average across sessions and participants, ranging from 25% to 41%). Viewed on our representation of the visual field of view, these opposite-phase voxels clustered outside or close to the edge of the stimulus field of view (Fig. 3A), in patterns that reproduced across sessions (Supplementary Figs. 1 and 2) and consistent with previously observed negative BOLD responses in cortical tissue neighboring stimulus-driven fMRI activations (Smith et al. 2004; Shmuel et al. 2006; Wade and Rowland 2010). The solid dark traces in Fig. 3A and Supplementary Fig. 1 outline our estimate of the cortical representation of the stimulus field of view, which leveraged these physiological negBOLD/posBOLD boundaries to refine estimates initially obtained from the surface-based registration of a retinotopic atlas (dashed dark circular outlines in Fig. 3A and Supplementary Fig. 1).

Large veins were clearly resolved in our 1 mm resolution maps of vein likeliness (red arrows in Fig. 3C’s bottom panel). They expectedly (Olman et al. 2007) colocalized with voxels with a low baseline BOLD signal (white arrows in Fig. 3C’s top panel), high temporal variability (data not shown) and large stimulus-driven modulation (blue arrows in Fig. 3C’s middle panel).

The dimensionality (number of voxels) of participants’ functional datasets was reduced independently within each of the two fMRI session repeats. In our example participant, selection for voxels (i) representing the stimulus’ field of view, (ii) significantly responding to the stimulus presentation, (iii) unlikely to contain veins, and (iv) most sensitive to stimulus orientation reduced the 4,697 gray-matter V1 voxel ROI to 1,111 in one session and 1,415 in the other. Across participants and sessions, the initial ROI contained 3,419 to 5,410 voxels and reduced to 570 to 1,415 voxels after feature selection (see Supplementary Table 1 for details).

### Decoding patterns of BOLD response delays and amplitudes

To assess whether different oriented visual gratings or plaid could produce different apparent delays of individual voxels’ BOLD response, we used the following logic: If we can predict the orientation profile of visual stimuli from the pattern of BOLD delay that they generate across the V1 cortical sheet, then these orientation profiles must modulate BOLD response delays in individual voxels. Importantly, to avoid circularity in predicting/classifying stimulus orientation (Kriegeskorte et al. 2009), both selection of relevant voxels (dimensionality reduction) and training of decoding spatial (SVM classifier) models relied on functional data from one “train” session while strictly reserving data from the other “test” session for cross-validation of decoding performances (see section Cross-Validation Between Sessions).

**Figure 4:**
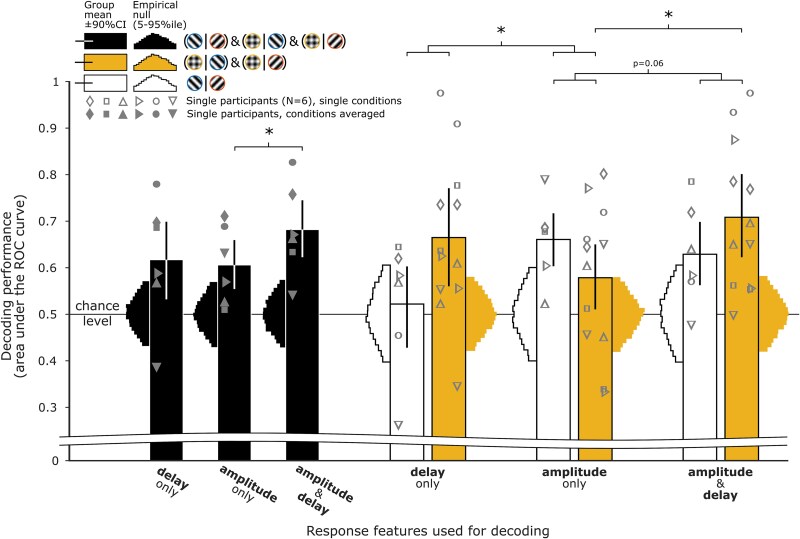
Decoding of V1 patterns of BOLD response amplitudes and apparent delays for pair-wise predictions of stimulus conditions. Dark bars show average prediction performances across all pairs of stimulus conditions. White bars show predictions of the orientation of the two gratings. Yellow bars average prediction performance across pairs comparing the plaid overlay stimulus to either grating. Sideway histograms show null distributions (5th to 95th percentiles) empirically derived from 8,192 random permutations of stimulus condition labels. Out of all decoding performances shown, only prediction of grating orientation from delay-only information did not significantly rise above chance (bar below the null distribution’s 95th percentile and error bar overlapping chance level). Error bars: 90% confidence intervals derived from 8,192 bootstrap resamples of *n* = 6 participants with replacement. ^*^: *P* < 0.05.

Both amplitude and apparent delay of BOLD responses could “alone” support accurate two-class prediction of stimulus conditions. Averaged across all pairs of stimulus conditions, AUC measures of decoding performance (Fig. 4, solid dark bars) rose to 0.62, respectively (delay-only: *t*_5_ = 2.1, one-sided *P* = 0.045; permutation test one-sided *P* = 0.003) and 0.61 (amplitude-only *t*_5_ = 3.1, one-sided *P* = 0.014; permutation test one-sided *P* = 0.006), significantly above chance (*H*_0_: AUC = 0.50). Interestingly, prediction using the Cartesian representation of response vectors—carrying both delay and amplitude information—offered the best performance, with an AUC of 0.68 (*t*_5_ = 4.5, one-sided *P* = 0.003; permutation test, one-sided *P* = 0). Adding delay information increased prediction performances (delay + amplitude vs amplitude only: *t* = −2.1117, one-sided *P* = 0.044). Cortical BOLD response apparent delays therefore “do” carry relevant information, which may inform on underlying neural processes.

### Stimulus-related modulation of the BOLD response delay

Converging evidence tends to associate neurally related BOLD delays with intracortical inhibitory processes (Muthukumaraswamy et al. 2009; Farivar et al. 2011; Muthukumaraswamy et al. 2012; Uhlirova et al. 2016a). We therefore tested whether single voxel’s apparent delay modulations in our experiment were more specifically driven by the neural suppression at play during the simultaneous processing of two orientations, ie during presentation of the plaid overlay stimulus. We found evidence for that in AUC decoding performances showing a significant interaction (*F*_1,5_ = 7.0, *P* = 0.046) between the type of stimulus prediction (plaid**|**grating vs −45°**|** + 45°) and the type of information it relied on (delay-only vs amplitude-only)—amplitude information supported the prediction of any type of stimulus whereas delay information supported only predictions involving plaid overlay stimuli, the condition putatively engaging higher levels of intracortical inhibition (Fig. 4, delay- and amplitude-only white and yellow bars). Finally, adding delay information to decoding based on amplitude information increased performance when plaids were involved (Fig. 4; trend for a type of information  ×  type of stimulus interaction: *F*_1,5_ = 5.9, *P* = 0.060; amplitude-only vs delay + amplitude for predictions involving plaids: *t* = 3.693, *P* = 0.014).

**Figure 5:**
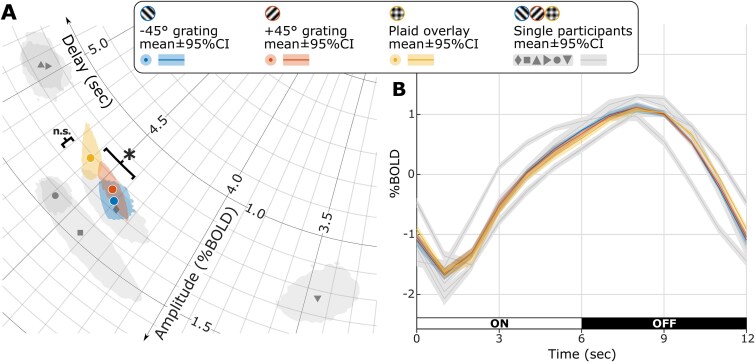
Group-level effect of stimulus condition on the hemodynamic response shape. A) Polar representation (as in Fig. 2A) of sinusoidal response vectors in V1, spatially averaged across selected voxels (feature selection steps A to D). Gray markers show the individual participant’s response averaged across stimulus conditions. Colored markers show the response to each stimulus conditions averaged across participants. B) Same data as in A), represented as the average BOLD time course through the ON–OFF stimulus cycle. In both A) and B), gray 95% error areas were bootstrapped from data with condition-means removed, whereas colored 95% error areas were bootstrapped from data with participant-means removed, respectively, highlighting the variability of within-participant estimates and of cross-condition effects.

Interestingly, when averaged across all selected voxels, responses to grating and plaid overlay stimuli were similarly distinct (Fig. 5): responses driven by the plaid overlay showed an apparent delay 160 ms longer (range: 78 to 327 ms) than those driven by gratings (plaid vs gratings: *t*_5_ = −4.3, *P* = 0.008; signed-rank = 21, *P* = 0.03; plaid vs −45° vs +45°: F_(2,10)_ = 6.8, *P* = 0.014), but all responses showed similar amplitudes (plaid vs gratings: Δ = 0.03%BOLD, *t*_5_ = −0.97, *P* = 0.375; signed-rank = 7, *P* = 0.563; plaid vs −45° vs +45°: *F*_(2,10)_ = 0.9, *P* = 0.432). Individual (subject- and condition-specific) delay estimates showed bootstrapped 95% CI ranging from widths of 0.106 to 0.615 s. Across participants, a stronger impact of plaid on response delay was only marginally related to weaker response amplitude to plaid compared to grating stimuli (Supplementary Fig. 3; Spearman’s R = −0.60, *P* = 0.24; Pearson’s R = −0.82, *P* = 0.05).

Together with the decoding results, this suggests that the orientation profile of visual stimuli affects the cortical pattern of BOLD response amplitudes. Importantly, increasing cross-orientation suppression by overlaying different orientations increases the apparent response delays across the V1 cortex.

## Discussion

Using optimized fMRI measures of the apparent delay of BOLD responses in the human early visual cortex, we found that V1 voxel patterns of delays are alone sufficient for predicting features of the driving visual stimuli, here the stimuli’s orientation profile. This challenges the common belief that temporal characteristics of fMRI responses are pure vascular artifacts. Instead, incorporating delay information in a decoding analysis of response patterns outperformed decoding based only on response amplitude. Moreover, response amplitudes and delays showed different characteristics. Overall, V1 response amplitudes were stable across all stimuli—both orthogonally oriented gratings and the contrast-matched plaid overlay of the two—but patterns of amplitudes differed. This is consistent with matched overall activity levels that, however, differently distributes in V1 tissues representing the stimuli’s orientation content (Haynes and Rees 2005; Kamitani and Tong 2005; Brouwer and Heeger 2011; O'Herron et al. 2016). For delays, the cortical patterns were indistinguishable across orientations when presented alone, but presenting them simultaneously as a cross-oriented plaid overlay delayed the overall V1 response by ~180 ms. Together, our findings suggest that neurally relevant information lies in the shape of hemodynamic signals. We speculate this information relates to decreased cortical excitation/inhibition ratios, such as during binocular cross-orientation suppression (Morrone et al. 1987; Suarez et al. 1995; Sengpiel and Vorobyov 2005). This has important implications for the use of noninvasive fMRI beyond the localization of active cortical tissues, opening the possibility of investigating the underlying neural computations with widely available clinical-grade MRI systems.

Several human studies (Bandettini et al. 1997; Hoge et al. 1999; Peck et al. 2001; Sadaghiani et al. 2009; Bartolo et al. 2011; Farivar et al. 2011; Thompson et al. 2014; Kashyap et al. 2018; Chen et al. 2021) reported changes in the shape of the hemodynamic response—within a given piece of brain tissue—upon different stimuli or task requirements not meaningfully expected to affect neural activity timing. Of note, Peck et al. (2001), found the BOLD HR in the supplementary motor area to show increasing delay with increasing level of the inhibitory control required for production of isometric forces with the fingers. Bartolo et al. (2011) reported increased early visual cortex BOLD delays in two macaque monkeys using stimuli like ours, along with different profiles of evoked spiking and local field potentials.

Farivar et al. (2011) more specifically investigated the impact of intracortical inhibition on the BOLD HR in the context of pathological interocular inhibition in amblyopia (Sengpiel et al. 2005; Sengpiel and Vorobyov 2005). Shmuel et al. (2002) had reported that negative BOLD dynamics differed significantly from positive BOLD, with the negative component being putatively related to suppression and/or inhibition. Building on that finding, Farivar et al. (2011) measured HR in response to stimulation of the suppressed amblyopic eye, compared to the response of the fellow eye, and showed longer delays upon brief monocular stimulations—they used the Shmuel et al. (2002) HR dynamics to support a spatial summation model of negative and positive BOLD components within a voxel interacting to generate a delayed HR. While pathological alterations of the cortical microvasculature in amblyopia may have confounded this result, they were able to show that modulating the functional inhibition with a dichoptic mask continuously presented to the inhibiting fellow eye further lengthened the delay, arguing against a purely vascular effect.

The above studies and ours support the hypothesis that different computations performed within the same piece of cortical tissue can lead to differently shaped HRs and that those involving more intracortical inhibition specifically increase the HR delay. Indeed, optogenetics studies have shown that activation of inhibitory interneuron alone can drive large hemodynamic responses (Anenberg et al. 2015, Uhlirova et al. 2016b) with time courses not matching those driven by the pyramidal neuron (Uhlirova et al. 2016b). Evidence linking HR delays with inhibition in humans remains scarce and indirect. More complete studies incorporating modulations of the neural substrate of inhibition, eg through brain modulation techniques (Stagg et al. 2009; Allen et al. 2014) or plasticity paradigms (Lunghi et al. 2015), are needed.

The suppressive effects of cross-oriented masks can begin subcortically (Freeman et al. 2002; Priebe and Ferster 2006) with contrast saturation in nonoriented thalamic neurons (Priebe and Ferster 2006). This was likely at play during the binocular presentation of our plaid stimulus. However, subcortical suppression is usually demonstrated using monocular stimulation (Freeman et al. 2002). On the other hand, dichoptic stimulation produces cross-orientation that, given its susceptibility to adaptation, is of cortical origin (Li et al. 2005). Suppression during our binocular stimulation, therefore, likely began subcortically and deepened in the V1 cortex (Walker et al. 1998; Li et al. 2005; Baker et al. 2007).

We cannot wholly exclude non-neural vascular effects. For example, noradrenergic (NA) tone has been shown to underlie the faster hemodynamic response to stimuli spanning a larger patch of the cortex—blocking of NA input reduces or eliminates this faster hemodynamic response (Bekar et al. 2012). Our plaid stimuli could be reasonably assumed to engage a larger patch of cortex as they would stimulate two sets of orientation columns instead of one, but we did not observe a speed-up of the hemodynamic latency—quite the opposite, we find the latency increases. Relatedly, the relationship between neural and vascular responses contains significant nonlinearities that may also implicate inhibitory systems (Sheth et al. 2004). Finally, as the BOLD response waveform has been shown to vary across cortical depth (Siero et al. 2011; Kashyap et al. 2018), the delay difference we measured may reflect different layer profiles of activation.

While we had no way of isolating or measuring from inhibitory interneurons, we base our inference/speculation of suppression-induced delay on previous work by Sengpiel and Vorobyov (2005) where cross-orientation suppression—using stimuli similar to ours—was shown to be mediated by the γ-aminobutyric acid (GABA) neurotransmitter. However, the relationship between vascular and neural responses is complex, and the bulk of the vascular control exerted by inhibitory neurons may operate through nitric oxide (NO) signaling (Drew 2019; Echagarruga et al. 2020; Krawchuk et al. 2020; Lee et al. 2020)—up to 80% according to Vazquez et al. (2018) in the mouse cortex. Even then, the possibility of a GABA-mediated mechanisms, as suggested by Vaucher et al. (2000), is not entirely excluded since Vazquez et al. (2018) only tested the function of GABA_A_ receptors and the nonsynaptic mechanism suggested by Vaucher et al. (2000) relies on GABA released in the extracellular space by axon varicosities and typically activating GABA_B_ receptors. With GABA_B_ receptors found on cerebral microvessels and capable of producing vasoconstriction (Fergus and Lee 1997), there is perhaps yet too little evidence to conclusively rule out a direct role of GABA in neurovascular signaling, and it therefore merits mention as a potential mechanism.

One limitation of this study is the lack of specificity to the actual temporal feature of the HR that is affected by stimulus condition. Indeed, stimuli can drive fast—up to 0.75 Hz—BOLD responses (Lewis et al. 2016) such that onset and offset transients (Gonzalez-Castillo et al. 2012) likely contributed to our measurements—eg they may underlie the broader positive lobe of the response relative to the negative one (Figs 1C, 2B, and  5B and Supplementary  Fig.  4). The delayed sinusoidal fit to overlay stimulus’ response could therefore stem, for example, from a smaller stimulus onset transient and/or a larger offset transient, or from complex modulations of other temporal features like onset time, slopes, and peak times. Those are, however, “by design,” not resolved in our experiment. Further measurements using event-related designs and/or longer blocks are needed to pinpoint the specific feature or features modulated.

Misspecification of the HR model can affect response amplitude estimation (Handwerker et al. 2004; Proulx et al. 2014). Response delay variations of the extent reported here should, however, have minimal impact on amplitudes estimated using a fixed-delay canonical hemodynamic response model—an amplitude underestimation of ~0.25% in event-related designs (see Supplementary Methods) and lower with longer stimulus blocs.

## Conclusion

Evidence is accumulating to show that temporal characteristics of hemodynamic signals such as their delay, when carefully measured and analyzed, can provide relevant information on the neural computations underlying fMRI activations. If a causal link were demonstrated, eg with the excitation/inhibition ratio, it would mean the latter could be noninvasively measured with widely available 3T fMRI scanners.

## Supplementary Material

2026_CerebralCortex_HRdelay_acceptedSupplements_bhag040(1)

## Data Availability

Intermediary data (preprocessed V1 voxels' timeseries) is available for download at https://doi.org/10.5281/zenodo.5183027. Analysis code is available at https://github.com/Proulx-S/HRdelay/releases/latest (DOI: https://doi.org/10.5281/zenodo.6568435).
