# Supplementary material for: BOLD response delays represent local cortical processing
Source: Cereb Cortex. 2026 Apr 22;36(4):bhag040. doi: 10.1093/cercor/bhag040 (PMC13099394; doi:10.1093/cercor/bhag040)
Supplement: 2026_CerebralCortex_HRdelay_acceptedSupplements_bhag040(1) [file 2026_cerebralcortex_hrdelay_acceptedsupplements_bhag040(1).docx]

Supplementary Materials

# For manuscript titled

BOLD Response Delays Represent Local Cortical Processing

# Authors

Sébastien Proulx^1*^ and Reza Farivar^1*^

# Affiliation

^1^Department of Ophthalmology & Visual Sciences, McGill University, Montreal, Canada

*[proulxs@stanford.edu](mailto:proulxs@stanford.edu); [reza.farivar@mcgill.ca](mailto:reza.farivar@mcgill.ca)

Montreal General Hospital, 1650 Avenue Cedar L11.409

Montréal, Québec, H3G 1A4, Canada

Tel: +1(514)-934-1934-44508

# Supplementary Methods

To assess the impact of unmodeled stimulus-specific HR delay variations on HR amplitude estimation, we fitted SPM12’s canonical HR function (all parameters set to default; https://www.fil.ion.ucl.ac.uk/spm/) to a 200ms-delayed version of the same function (code for this available online DOI: 10.5281/zenodo.7058825). This yielded a coefficient of 0.9976, meaning the response amplitude is underestimated by 0.24%.

# Supplementary Table

|  | Number of voxels after successive dimensionality reduction steps, averaged across sessions (feature selection steps) | | | | |
| --- | --- | --- | --- | --- | --- |
| Participants | Initial ROI | & within cortical representation (A) | & stimulus driven (B) | & non-vein (C) | & sensitive to stimulus condition (D) |
| 02jp | 3,419 | 2,565.5 | 1,833.5 | 1,467.0 | 1,173.5 |
| 03sk | 4,697 | 2,945.0 | 1,973.5 | 1,579.0 | 1,263.0 |
| 04sp | 4,747 | 1,891.5 | 1,257.5 | 1,006.0 | 804.5 |
| 05bm | 5,410 | 2,801.0 | 1,735.0 | 1,388.0 | 1,110.5 |
| 06sb | 4,057 | 2,370.0 | 1,683.5 | 1,346.5 | 1,077.0 |
| 07bj | 3,793 | 2,233.5 | 952.5 | 762.0 | 609.5 |
| Average | 4,354 | 2,468.8 | 1,573.6 | 1,258.1 | 1,006.3 |

**Supplementary Table 1.** Summary of dimensionality reduction, from feature selection steps A to D. From left to right, the number of selected voxels diminishes as successive feature selection steps are applied.

# Supplementary Figures


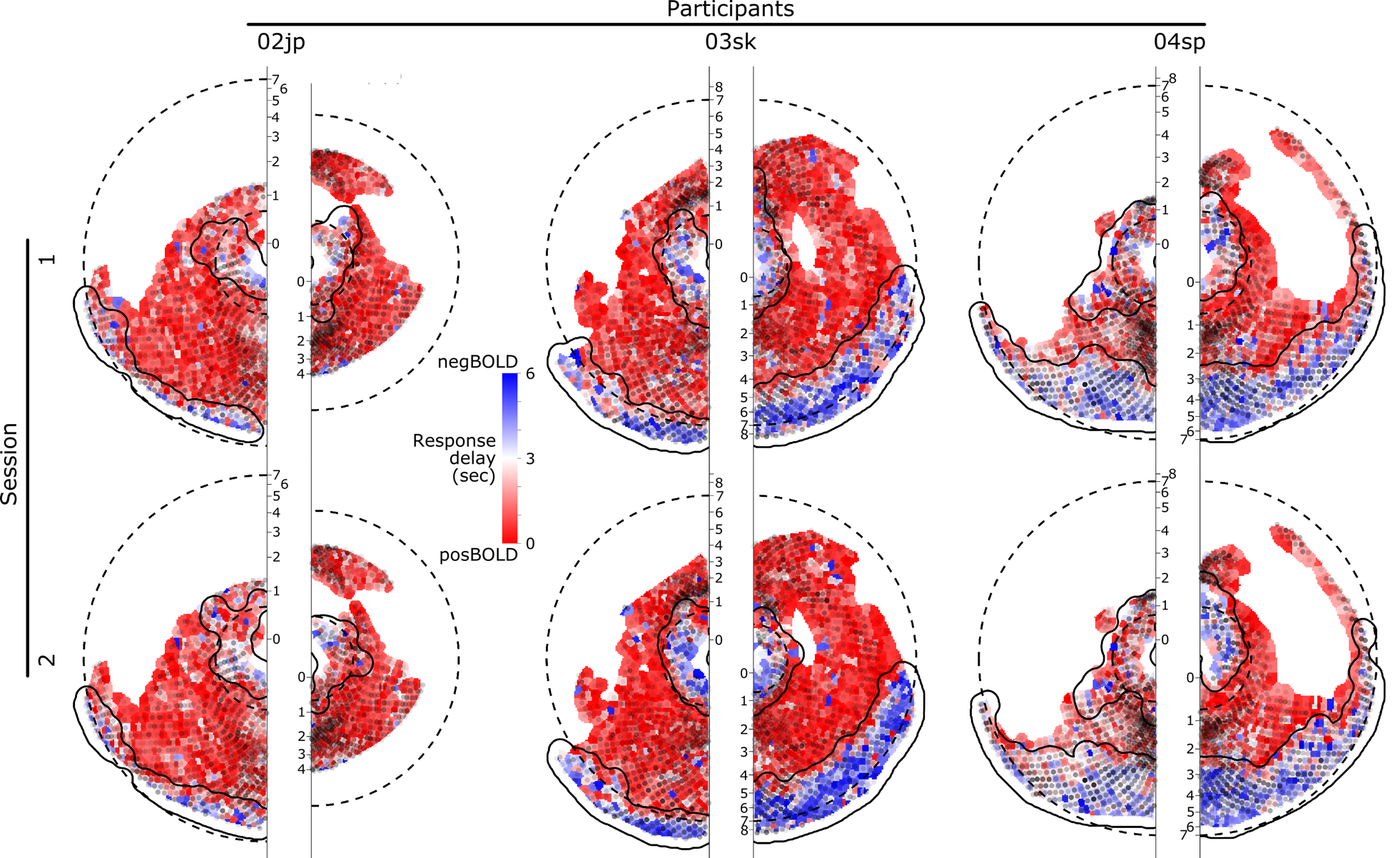


**Supplementary Figure 1.** BOLD response polarity mapped to the scaled visual field for each hemisphere, participant and session. Same convention as Figure 3 of the main text. Scaling of the visual field affected only the radial axis, relying on linearization of the…


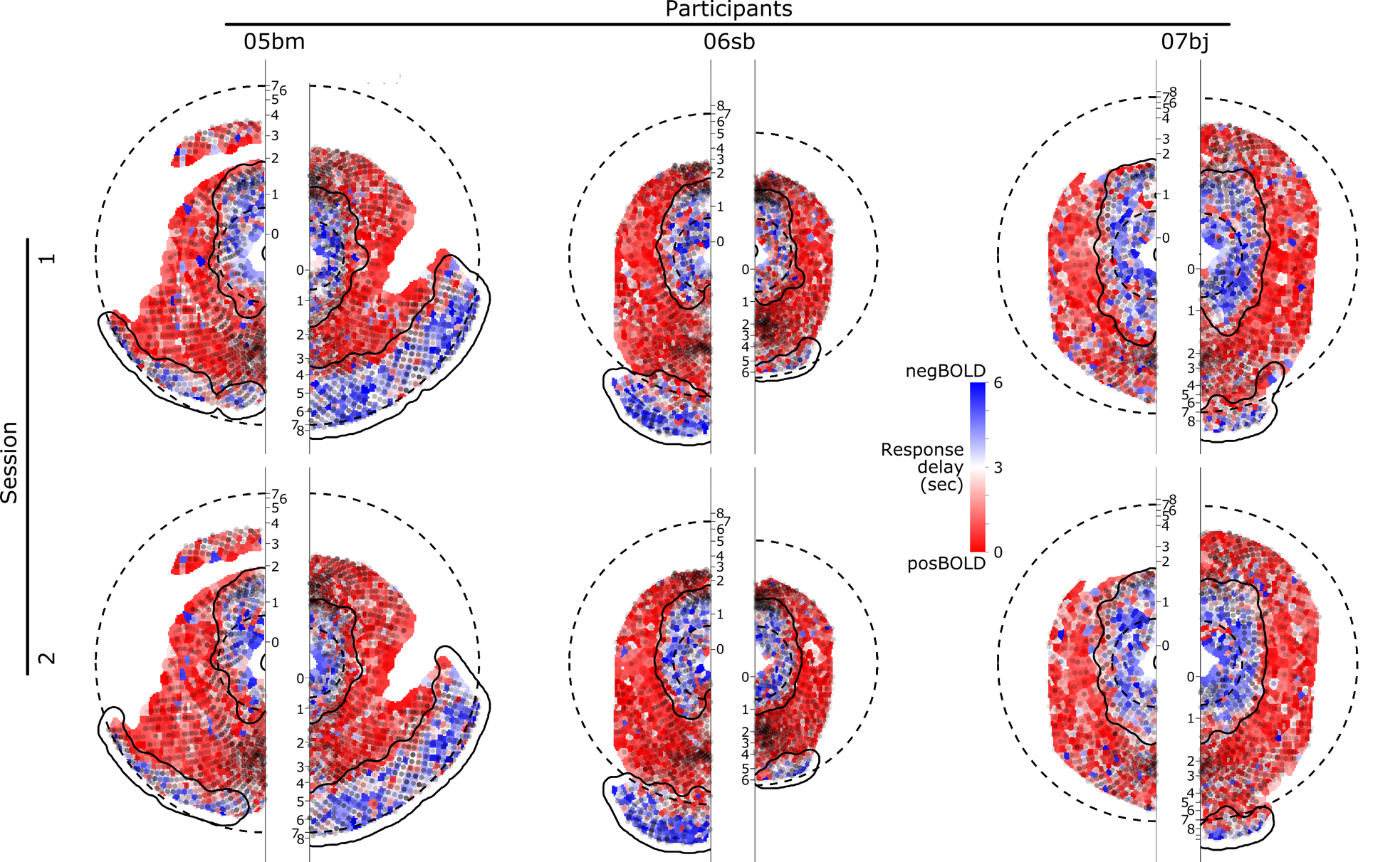


**Supplementary Figure 1. *cont.*** …cumulative distribution function of ROI voxel’s eccentricities to counteract cortical magnification and allow visualization on a more homogeneously represented (uniform voxel density) visual field. Scaling was performed…

**Supplementary Figure 1. *cont.*** …independently for each participant’s hemisphere. It was however the same across sessions since it ultimately depended on the geometry of the fMRI imaging grid, which was very similar by design across session and made the same after preprocessing. Note that polarity maps, on the other hand, are based on independent data across sessions and yet show remarkable similarities within all participants. The heuristic algorithmic approach aimed at outlining the negBOLD regions surrounding the cortical representation of the annular stimulus field-of-view. It began with extracting the contours of the smoothed polarity map at a value mid-way between negBOLD and posBOLD. It selected the contours that, after slight inflation, overlaps the outside of the visual stimulus field-of-view as defined from the probabilistic retinotopic atlas (dashed outlines). The selected contours generally outlined multiple small areas of negBOLD, which were merged into generally two areas (one for the inner surround and the other for the outer surround of the stimulus field-of-view) by successive inflation and deflation. The resulting contours were inflated again such that the non-negBOLD area now constitutes a conservative estimate of the region responding with posBOLD to the stimulus, the latter being taken as a functional estimate of the cortical representation of the stimulus field-of-view. As this did not catch all clearly negBOLD areas, the process was repeated using heavier smoothing of the map and replacing the retinotopic estimate of the representation of the stimulus’ field-of-view by that provided by the first iteration. This second pass provided an even more conservative definition of the stimulus’ representation by incorporating more negBOLD speckled areas that tended to neighbor the clearly negBOLD areas.


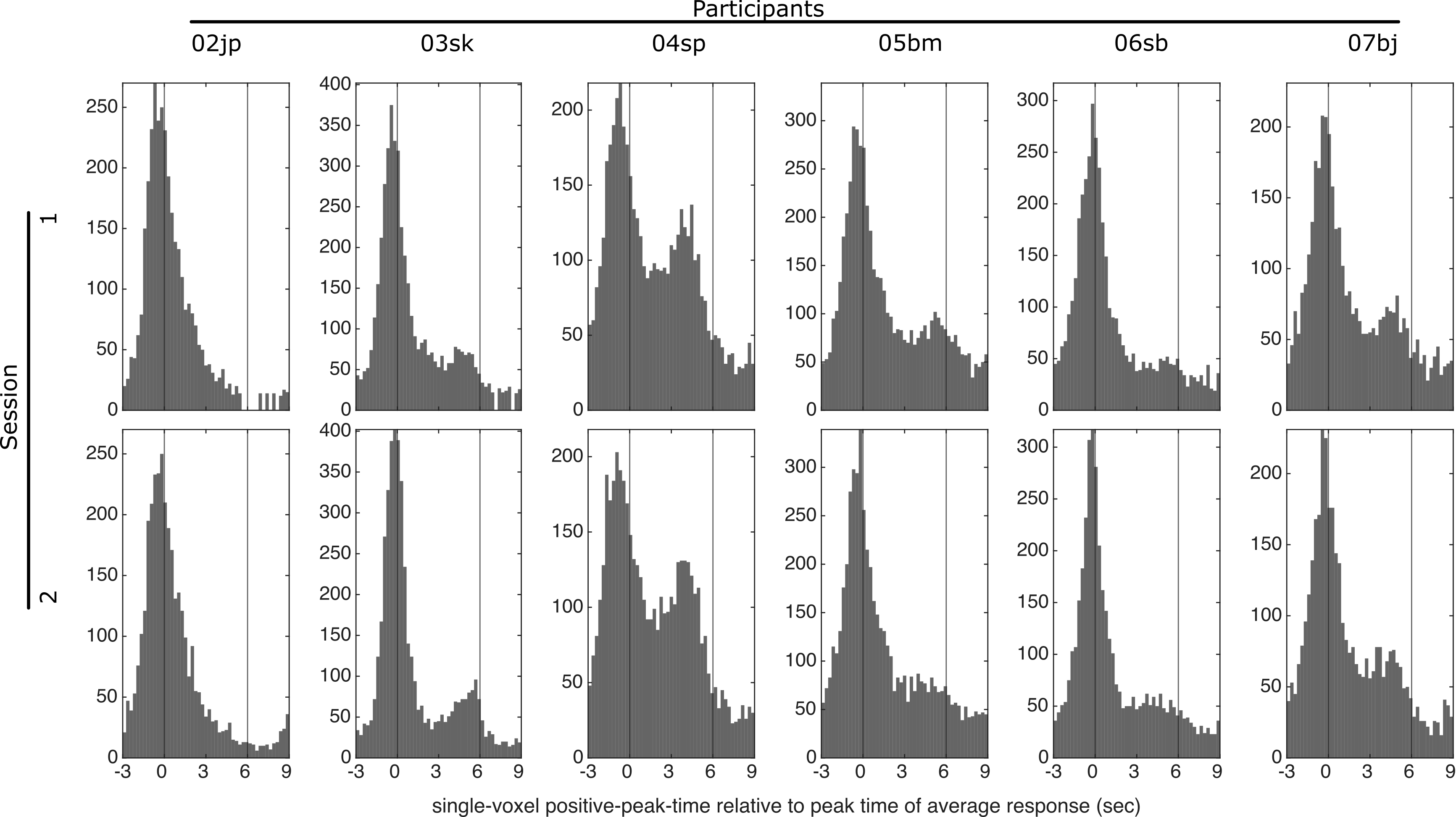
 **Supplementary Figure 2.** Histograms of the positive-peak-time delay of single-voxels’ sinusoidal fitted response, relative to the positive-peak-time of the V1 ROI average response (vertical lines at 0s; vertical lines at 6s show the half-cycle delay of an opposite-polarity response). Same as the inset of Figure 3A, but for all participants and sessions.

**
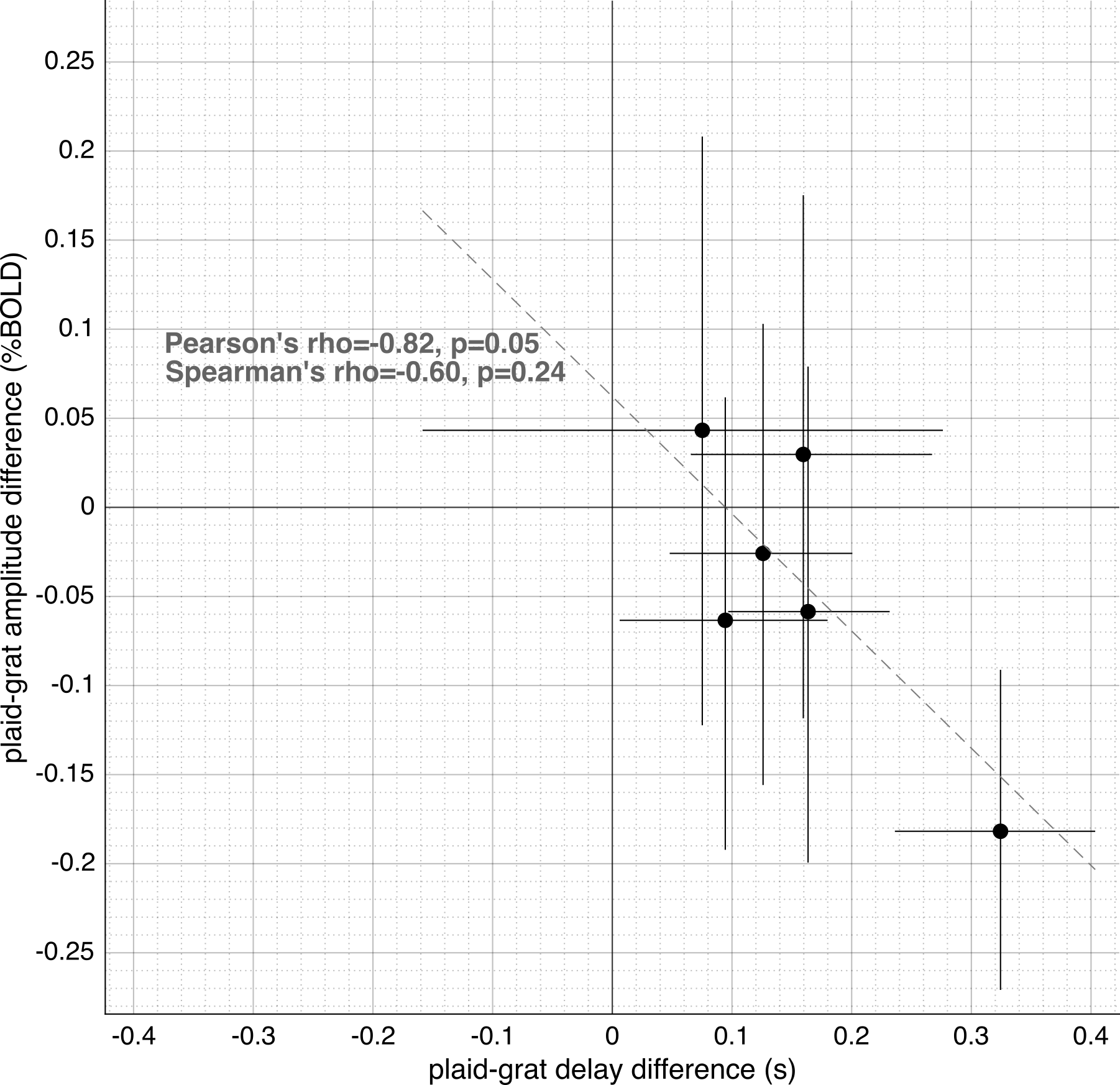

Supplementary Figure 3.** Relation between the effect of the plaid stimulus on response amplitude and delay relative to grating stimuli. The trend toward a relation is driven by a single participant. Each point is a participant. 95%CI bootstrapped across runs.


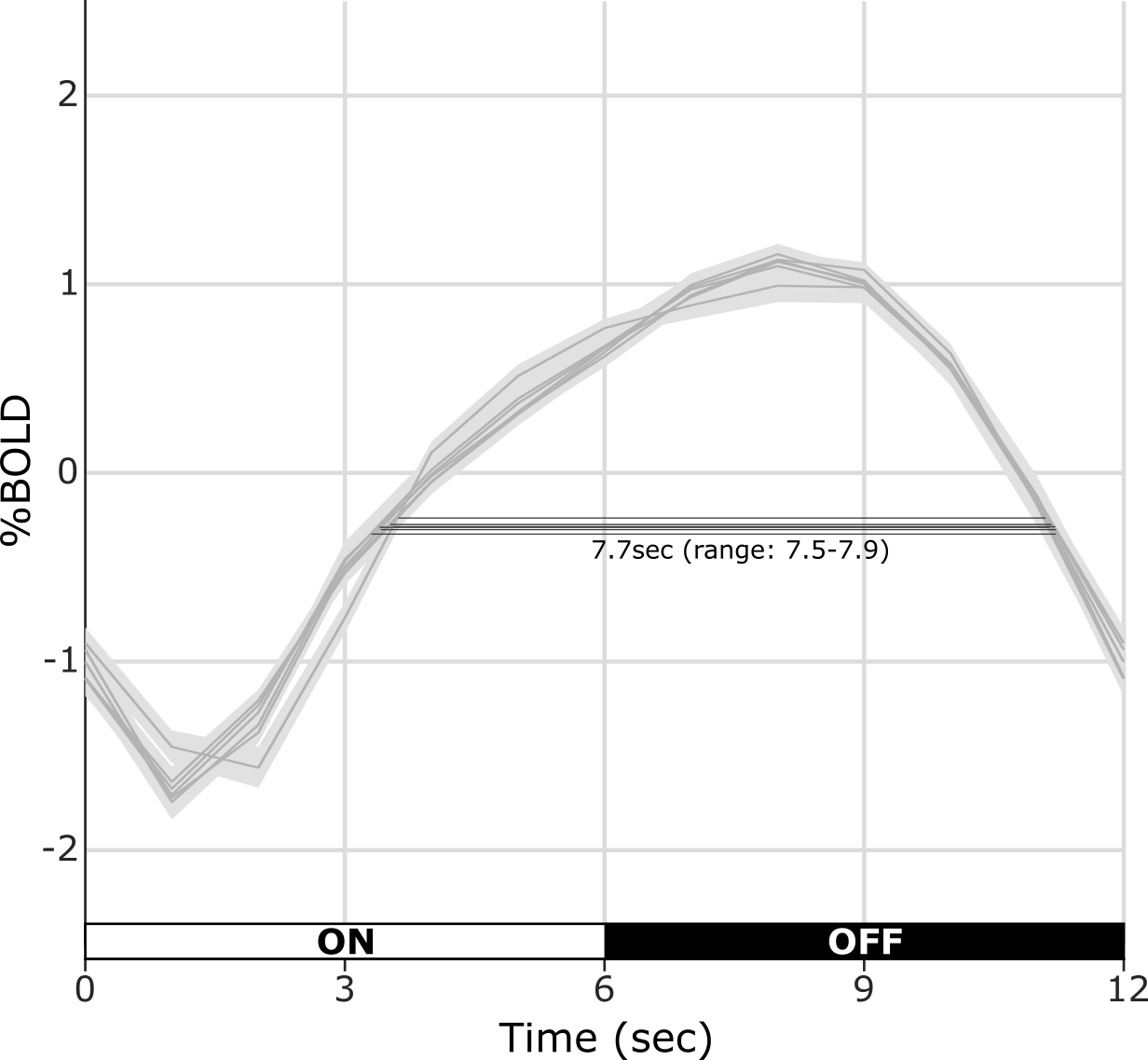


**Supplementary Figure 4.** Model-free estimate of stimulus responses, averaged across V1 voxels representing the stimulus FOV (feature selection step A) in each participant, normalized to the same sinusoidal response delay and amplitude for visualization of the overall shape. Horizontal lines indicate the width of the positive lobes at half the peak-to-peak amplitudes. Shaded areas: 95% CI bootstrapped across all runs.
